# Comparison and Optimization of Reactive Dyes and Coating Performance on *Fraxinus mandshurica* Veneer

**DOI:** 10.3390/polym10121302

**Published:** 2018-11-24

**Authors:** Xiaoxing Yan, Xingyu Qian, Rong Lu, Tetsuo Miyakoshi

**Affiliations:** 1 Co-Innovation Center of Efficient Processing and Utilization of Forest Resources, Nanjing Forestry University, Nanjing 210037, China; yanxiaoxing@njfu.edu.cn; 2 College of Furnishings and Industrial Design, Nanjing Forestry University, Nanjing 210037, China; qianxingyu@njfu.edu.cn; 3 Department of Industrial Chemistry, School of Science and Technology, Meiji University, 1-1-1 Higashi-Mita, Tama-ku, Kawasaki-shi 214-8571, Japan; miya@meiji.ac.jp

**Keywords:** *Fraxinus mandshurica* veneer, reactive dyes, waterborne coatings

## Abstract

In this study, *Fraxinus mandshurica* veneer was dyed with reactive brilliant red X-3B, black KN-B and blue K-3R dyes. The dye concentration, bath ratio and dyeing time were selected for an orthogonal experiment. Analysis of variance showed that the dye concentration had the greatest effect on the dye uptake of *F. mandshurica* veneer. In the independent experiments, dye uptake increased at first and then decreased with increasing dye concentration; the chromatic aberration increased with the dye concentration and then remained steady. The infrared spectra were used to examine the dyeing behaviors before and after dyeing and the binding form between reactive dyes and *F. mandshurica* veneer was analyzed. Based on the optimization of process parameters, the optimal dyeing condition was considered to be 75 °C, the dye concentration to be 0.5–1.0%, the dyeing time to be 60 min and the bath ratio to be 20:1. The dye uptakes of reactive brilliant red X-3B, black KN-B and blue K-3R dyes were 75.0–75.4%, 50.0–64.6% and 32.0–66.0%, respectively. The chromatic aberration of *F. mandshurica* veneer dyed with reactive brilliant red X-3B, black KN-B and blue K-3R dyes was 53.0–59.0, which was a significant increase. After dyeing, the hardness and impact strength of the waterborne coating on the dyed *F. mandshurica* increased but adhesion was reduced. The coating films produced a matte glossiness.

## 1. Introduction

There are various kinds of dyes for wood, acid, alkaline, reactive, direct, natural biological, etc. [1]. Among them, acid dyes, which mainly color wood lignin, are most widely used [2]. However, they have defects such as easy fading and poor water resistance. The related research showed that the adsorption of dye molecules to wood was a physical adsorption when acid dyes were used [3]. When reactive dyes were used, they penetrated the interior of the wood and reacted with the wood components to form a covalent bond [4]. Therefore, compared with acid dyes, the stability of wood dyed with reactive dyes was higher [5]. In the dyeing process, changes in different factors such as dyeing time, bath ratio and dye concentration usually produced different effects [6]. High concentrations of dye molecules in liquid increased the viscosity, thus affecting the adsorption and permeability.

Waterborne coatings are widely used in many industries because of their green, non-polluting characteristics [7]. Due to the advantages of containing few volatiles, no pollution and high safety, waterborne coatings for wood are the main research object. High gloss can have a negative effect on people’s eyesight, making the low gloss of waterborne wood coatings increasingly popular [8]. So far, many researchers have studied the dyeing process of poplar [9] but fewer have studied the effects of dyeing on subsequent finishing processes. Although *Fraxinus mandshurica* is a material commonly used for the veneer of high-grade furniture [10], there are few studies on reactive dyeing and waterborne finishing of *Fraxinus mandshurica*. In this paper, the dyeing properties of three kinds of reactive dyes including reactive brilliant red, reactive blue and reactive black dyes on *F. mandshurica* were examined by an orthogonal test and independent experiments. Based on the results of the dye uptake and chromatic aberration, the effects of different reactive dyes on the properties of waterborne coatings on the *F. mandshurica* were evaluated and optimized and a preliminary discussion on the chemical and physical properties of dyeing veneers was also carried out.

## 2. Materials and Methods

### 2.1. Materials

**Figure 1:**
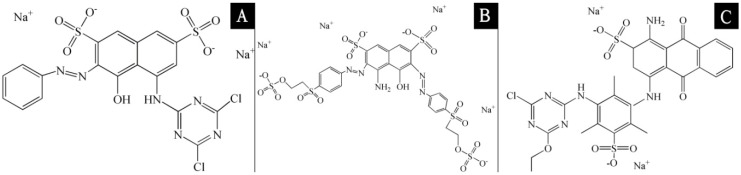
The structures of (**A**) reactive brilliant red X-3B dye, (**B**) reactive black KN-B dye and (**C**) reactive blue K-3R dye.

*F. mandshurica* veneer (uniform material color, 40 mm × 40 mm × 3 mm) came from Yihua Lifestyle Technology Co. Ltd., Shantou, China. Reactive brilliant red X-3B dye, reactive black KN-B dye, reactive blue K-3R dye, sodium sulfate (Na_2_SO_4_) and sodium carbonate (Na_2_CO_3_) were supplied by Jiangsu Zhengyang Dyestuff Technology Co. Ltd., Wuxi, China. The structures of the reactive brilliant red X-3B, reactive black KN-B and reactive blue K-3R dyes are shown in Figure 1 where distilled water was used as the solvent for these dyes. Waterborne coatings were supplied by Yihua Lifestyle Technology Co. Ltd., Shantou, China. Waterborne coatings consisted of acrylic copolymers supported by water (the content was 90.0%), dipropylene glycol methyl ether (the content was 2.0%) and dipropylene glycol butyl ether (the content was 8.0%).

### 2.2. Dyeing and Coating Test

The experimental process was as follows: ash veneer → immersion in dyeing solution → constant temperature heating → dyeing → veneer fixing treatment → repeated distilled water washes until no fading → veneer drying → coating three times.

Because the dye concentration, dyeing time and bath ratio were the main factors effecting dyeing in this study, they were selected as the three parameters. A three-factor, two-level L_4_ (2^3^) orthogonal test scheme was designed and is summarized in Table 1. The first level of the dye concentration was 1.0% and the second was 5.0%. The first level of the bath ratio was 20:1 and the second was 30:1. The first level of dyeing time was 60 min and the second was 120 min. According to the requirements of the liquor ratio, Na_2_SO_4_ and Na_2_CO_3_ concentrations were fixed at 2.0% and the temperature of dyeing was controlled at 75 °C. It was reported that high temperatures are usually recommended for best dye penetration and dyeing effect. The controlled temperature, 75 °C, did not lead to hydration of functional groups in reactive dyes [11]. According to the requirements of the dyes, Na_2_SO_4_ and distilled water were mixed to the desired concentrations. The bath ratio was the weight ratio of the dye liquor to the dyeing specimen. The weight of the *F. mandshurica* veneer for each time dyed was about 3.5 g. According to the bath ratio, the quantity of the dye liquor was about 70–105 g. The *F. mandshurica* veneers were placed in the heated dye liquor and Na_2_CO_3_ was added after the stipulated dyeing time to solidify color processing. The dyeing solution was cooled to 60 °C and kept at a constant temperature. The fixing agent Na_2_CO_3_ was added, stirred evenly and placed for 30 min before the veneer fixing treatment was finished. After fixing the color, the dyed *F. mandshurica* veneer was removed from the thermostat water tank and the specimen was cleaned with distilled water until the water had no discoloration. Then, the stained specimens were placed in the oven at 80 °C for 15 min to dry. The wash water solution was added to the dyeing wastewater to the same quantity as before dyeing (70–105 g) for determination of the dye uptake. The results of the orthogonal test were analyzed, calculating for range and variance and independent experiments were carried out with the most important factors.

The waterborne coatings were sprayed onto the dyed veneer of *F. mandshurica* by an airbrush (Guangzhou Zhongtian Electrical Equipment Co. Ltd., Guangzhou, China). The coating was naturally dried for 3 h and then, using 1000 grit sandpaper, the waterborne coating was sanded and a dry cloth was used to wipe off the dust. The spray process needed to be repeated three times. The thickness of the waterborne coating was about 40 μm.

### 2.3. Characterization

The dye uptake was determined by the spectrophotometric method [12]. The specific method was as follows: The absorbance of dye liquor before dyeing (A_0_) and the mixed liquor of waste liquid and cleaning liquid after dyeing (A_1_) were measured using a 752N type UV-Vis spectrophotometer and calculated according to the following formula:

C (%) = (A_0_ − A_1_)/A_0_ × 100% (1)

In Formula (1), C is dye uptake. The higher the dye uptake, the more dye wood absorbed in the process of dyeing. The HP-2136 chromatic aberration meter was used to directly measure the lab value of the specimen before and after dyeing. The chromatic aberration was measured at three points and the average value was obtained. The differences in color before and after dyeing were represented by ΔL, Δa and Δb, where ΔL is the difference in brightness, Δa is the red and green index difference and Δb is the yellow and blue index difference. Therefore, the chromatic aberration (ΔE) was calculated according to the following formula [13]:

(2) $$\Delta E=\sqrt{{\left(\Delta L\right)}^{2}+{\left(\Delta a\right)}^{2}+{\left(\Delta b\right)}^{2}}$$

The greater the chromatic aberration, the better the dyeing effect. The greater the ΔL was, the more the lightness decreased. The greater the Δa was the more red color the dyed material presented. A greater Δb indicates that the dyed material tended to be yellow. The infrared spectrum before and after dyeing was tested using a VERTEX 80V infrared spectrum analyzer (Bruker, Germany). The strength of the waterborne coatings on the dyed veneer of *F. mandshurica* was measured by a QCJ impactor. A QFZ-II coating adhesion tester was used to test the adhesion of the coating on the dyed veneer. Both the impactor and adhesion tester were produced by Tianjin Jingkelian Material Testing Machine Co. Ltd., Tianjin, China. The 6H–6B pencil test was used to test the hardness of the coating on the dyed veneer of *F. mandshurica*. For this test, the angle between the pencil and the coating was 45° and the pencil scratched under 1.0 kg load. The hardness of the pencil (6H, 5H, 4H, 3H, 2H, 1H, HB, 1B, 2B, 3B, 4B, 5B and 6B) was used to determine the hardness of the coating when there was initial scaring on the coating. A BGD512-60° gloss meter, produced by Suzhou Essen Instrument Equipment Co. Ltd., Suzhou, China, was used to measure the gloss of the waterborne coatings on the dyed veneer of *F. mandshurica*. A cone hole with a top angle of 120° was drilled on the coatings. The cone hole was drilled in the field of view of the microscope and the wall of the hole was imaged with the microscope magnified 40 times. The coating part of the bus bar perpendicular to the microscope’s main axis was read out. The length of the coating part of the bus bar was measured. According to the trigonometric function relation, it was evident that the thickness of the coating was half of the length of the coating part of the bus bar. The arithmetic mean of three points was used to evaluate the waterborne coating thickness.

## 3. Results and Discussion

### 3.1. Orthogonal Test

The dye uptake and chromatic aberration of the dyeing veneer of *F. mandshurica* were affected by three factors, dye concentration, dyeing time and bath ratio, and the effects on the dye uptake were obvious according to the comparative analysis of range. The effects of the above three factors on the dye uptake of dyed veneers are summarized in Table 1. The range values and variances corresponding to the same factors are calculated in each column in Table 1, the greater the range value and variance, the more significant the effect of the factor. In comparison with range value and variance, dye concentration had the most significant impact on the dye uptake of the dyeing veneer of *F. mandshurica*, while the effect of the other two factors was small. After optimization, the dye uptake and chromatic aberration of dyed veneer were optimized. Based on Table 1, it was obvious that the dye uptake of samples 1_Red_, 1_Black_ and 1_Blue_ were higher than those of other dye samples. Therefore, in the next optimization experiment the dyeing time was fixed at 60 min and the bath ratio was fixed at 20:1.

Based on the results from Table 1, chromatic aberrations of four sample sets were tested in orthogonal experiments. This is summarized in Table 2, Table 3 and Table 4. The parameters L, a and b are the color values of the *F. mandshurica* veneer before being dyeing. The parameters L’, a’ and b’ are the color value parameters of *F. mandshurica* veneer after dyeing. The different colors of *F. mandshurica* veneer before and after dyeing with the reactive brilliant red X-3B dye, reactive black KN-B dye and reactive blue K-3R dyes are represented as ΔL, Δa and Δb. Chromatic aberration of *F. mandshurica* veneer before and after dyeing is represented as ΔE. Table 2, Table 3 and Table 4 demonstrate that after dyeing with reactive brilliant red X-3B dye, reactive black KN-B dye and reactive blue K-3R dye, a great change of chromatic aberration of *F. mandshurica* was observed. The values of chromatic aberration were large, 54–60, suggesting that the veneers of *F. mandshurica* after dyeing with the reactive brilliant red X-3B dye, reactive black KN-B dye and reactive blue K-3R dye were all obviously stained. The negative ΔL (Table 2, Table 3 and Table 4) indicated that the lightness decreased. Table 2 shows that the Δa was positive, indicating that the color of dyed *F. mandshurica* veneer was red. Table 3 and Table 4 show the Δa as being negative, indicating that the color of dyed *F. mandshurica* veneer was green.

### 3.2. Effects of Dye Concentration

**Figure 2:**
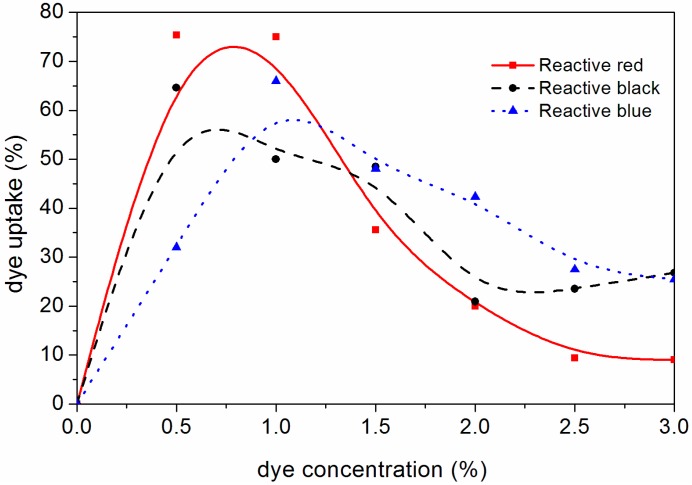
Effects of reactive brilliant red X-3B, black KN-B and blue K-3R dye concentrations on dye uptake.

According to the orthogonal test, the bath ratio was 20:1 and the dyeing time was 60 min. Based on the orthogonal experiment design, the dye concentrations were 0%, 0.5%, 1.0%, 1.5%, 2.0%, 2.5% and 3.0%, respectively, in the independent experiments to evaluate the dye uptake and chromatic aberration. Figure 2 shows the concentration of reactive brilliant red X-3B dye, reactive black KN-B dye and reactive blue K-3R dye on the dye uptake of dyed *F. mandshurica* veneers. It can be seen in the dyeing experiments of the above three kinds of dyes that dye uptake of the dyed veneer first increased and then decreased with the increase in dye concentration. When the concentration of reactive red dye increased from 0 to 1.0%, the dye uptake increased from 0 to about 75.0%. This is because when the dye concentration increased, the number of dye molecules in the liquid increased, thus increasing the contact force of veneer of *F. mandshurica* fiber surface adsorption [14]. However, when the reactive brilliant red X-3B dye concentration increased from 1.0% to 3.0%, the adsorption of dye molecules on fibers of *F. mandshurica* veneer would gradually reach saturation and, therefore, the dye uptake would decline [15]. Figure 2 shows that the dye uptake was improved in the dyeing process when the concentration of reactive brilliant red X-3B dye was 0.5–1.0%.

When the concentration of reactive black KN-B dye increased from 0 to 0.5%, the dye uptake was the highest. When the concentration of the reactive black dye was 0.5–2.0%, the dye uptake decreased. With the increase of reactive black dye concentration, the contact probability of reactive black dye and veneer of *F. mandshurica* fibers increased when the reactive black dye concentration increases. The solution was too viscous when the concentration of reactive black dye increased to 0.5%; this reduced the penetration of dye molecules so the dye uptake was reduced. When the reactive black dye concentration was further increased to 3.0%, the dye uptake increased slightly but remained at a lower value. When the concentration of the reactive black dye was 0.5–1.0%, the dye uptake of reactive black KN-B was improved.

When the concentration of the reactive blue K-3R dye was 0.5–1.0%, the dye uptake rose. However, the dye uptake decreased with further increases in the reactive blue K-3R dye concentration. This was because with the increase in reactive blue K-3R dye concentration, the reactive blue K-3R dye molecules aggregated in the *F. mandshurica* veneer, resulting in reduced liquidity, which plugged the wood cell walls of the hole and reduced the permeability of reactive blue K-3R dye [16]. Therefore, the dye uptake was improved in the reactive blue K-3R dyeing process when the reactive blue K-3R dye concentration was 1.0–1.5%.

**Figure 3:**
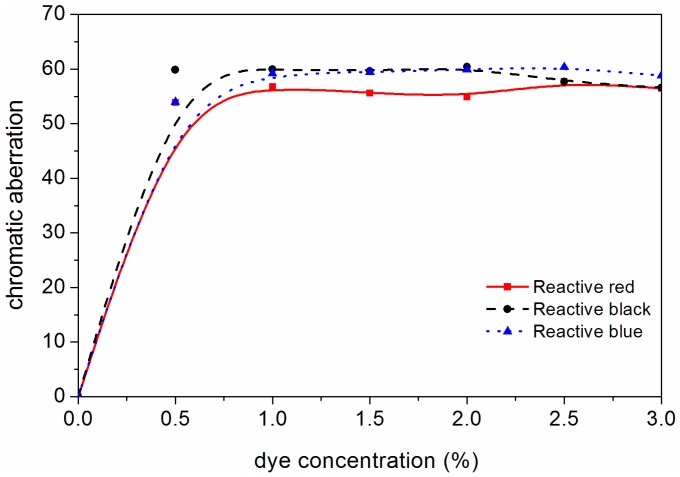
Effect of reactive brilliant red X-3B dye, reactive black KN-B dye and reactive blue K-3R dye concentrations on chromatic aberration.

Figure 3 shows a trend of chromatic aberration with the three kinds of dye. In the dyeing experiments of the above three dyes, with the dye concentration increased from 0 to 1.0%, the chromatic aberration value of *F. mandshurica* veneer increased sharply from 0 to about 60. Then, when the reactive brilliant red X-3B dye, reactive black KN-B dye and reactive blue K-3R dye concentration increased from 1.0% to 3.0%, the veneer chromatic aberration values increased slowly and tended towards stable values. Considering the production cost and energy consumption, the optimal concentration of three kinds of dyes is 0.5–1.0%.

### 3.3. Infrared Spectrum Analysis of Dye Concentration on Properties of F. mandshurica Veneer

**Figure 4:**
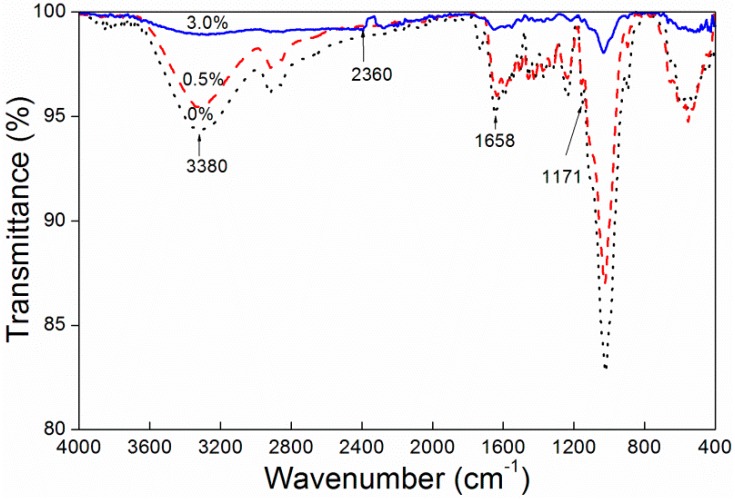
IR spectra of *F. mandshurica* veneer after dyeing with different concentrations of reactive red.

**Figure 5:**
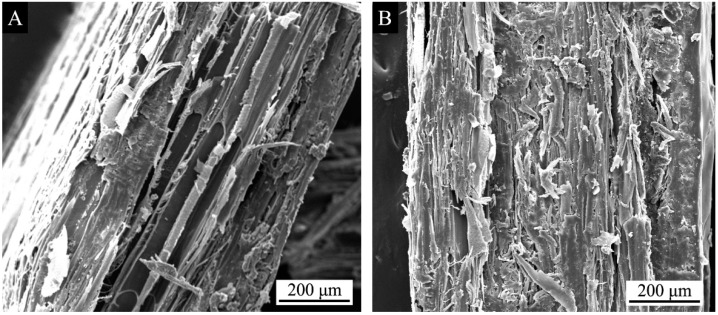
Side SEM of *F. mandshurica* veneer after reactive red dyeing at different concentrations of (**A**) 0.5% and (**B**) 3.0%.

Figure 4 shows the infrared spectrum of unstained *F. mandshurica* veneer and dyed *F. mandshurica* veneer of 0.5% and 3.0% concentration of reactive red dye, respectively. The assignment of peaks is summarized in Table 5. The band at 3380 cm^−1^ is the –OH stretching vibration of *F. mandshurica*. It can be seen that when the dye concentration was 0.5%, it showed a similar 3380 cm^−1^ band in both dyed and undyed samples, indicating that when the dye concentration was 0.5% the combination of reactive dyes and *F. mandshurica* was in the form of physical adsorption [17]. The peak at 3380 cm^−1^ slightly decreased suggesting that a chemical reaction of –OH and dye molecules occurred between the dye liquor and *F. mandshurica* in the dyeing process [18]. In the 3.0% reactive brilliant red X-3B staining of *F. mandshurica* the –OH stretching vibration at 3380 cm^−1^ disappeared, possibly due to reactive brilliant red dye coverage of the –OH group in the *F. mandshurica* or due to the reaction of reactive brilliant red and –OH. When the *F. mandshurica* was dyed by a 3.0% concentration of reactive brilliant red X-3B, a new peak 2360 cm^−1^ was produced for the –CN stretching vibration [19]. After the dye treatment, N–H–O absorption at 1658 cm^−1^ of *F. mandshurica* decreased. After the 3.0% dye concentration treatment, the C–O–C bond (1171 cm^−1^) absorption band was enhanced, indicating that the dehydration reaction between the –OH group reactive brilliant red and the hydroxyl groups in *F. mandshurica* had occurred and formed a C–O–C covalent bond [20]. Therefore, the combination of reactive brilliant red dye and *F. mandshurica* was in two forms in the dyeing process, physical combination and chemical reaction, and that in the test concentration (0–3.0%) range, with the increase of reactive brilliant red dye concentration, the chemical reaction between the reactive brilliant red dye and *F. mandshurica* was more obvious. This indicated a better combination [21]. With the increase of the reactive brilliant red concentration (Figure 5), the reactive brilliant red had a very uniform coverage of *F. mandshurica* and the reactive brilliant red was basically filled in the fibrous tissue of *F. mandshurica*.

**Figure 6:**
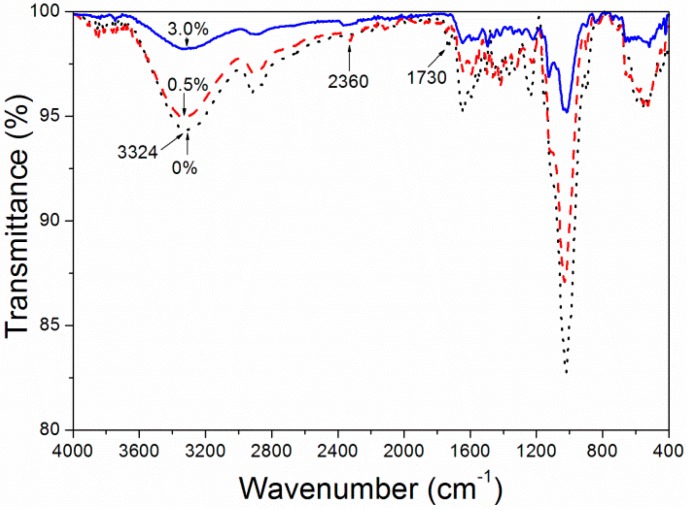
IR spectra of *F. mandshurica* veneer after dyeing with different concentrations of reactive black.

Figure 6 is the infrared spectra peak graph of unstained *F. mandshurica*, 0.5% and 3.0% reactive black KN-B dye concentration stain samples. The attribution of the infrared spectra band is summarized in Table 5. When the reactive black KN-B dye concentration was 0.5%, there was a gentle peak in the band at 1300–1500 cm^−1^ and a new band at 2360 cm^−1^ of the –CN stretching vibration was produced. This was formed by the reaction of carbonyl groups in *F. mandshurica* and –NH_2_ groups in reactive black dye [22,23]. When the reactive black KN-B dye concentration was 3.0%, it was obvious that the absorption band of absorption of N–H at 3324 cm^−1^ was weakened and that the carbonyl groups at 1730 cm^−1^ had disappeared due to the reaction of carbonyl groups in *F. mandshurica* and –NH_2_ groups in reactive black dye. With the increased concentration of reactive black KN-B dye, the reaction between the dye and *F. mandshurica* was more obvious, indicating that the combination was firmer.

**Figure 7:**
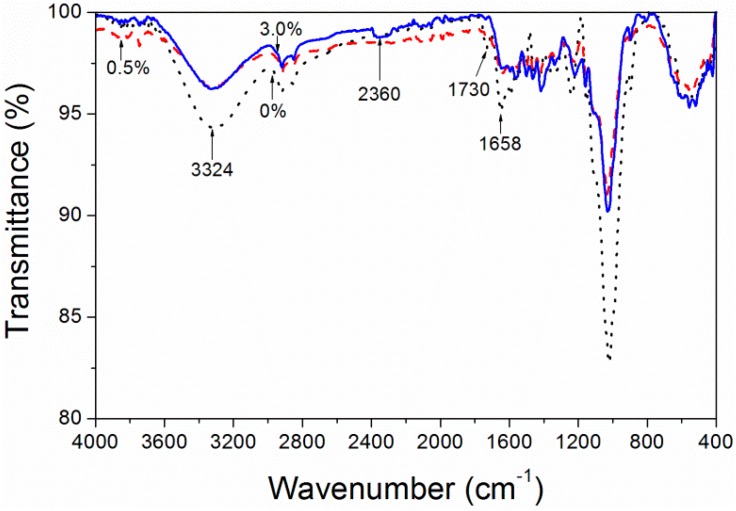
IR spectra of *F. mandshurica* veneer after dyeing with different concentrations of reactive blue.

Figure 7 is a graph of the infrared spectra peak of unstained processing *F. mandshurica* and the dyeing samples with 0.5% and 3.0% reactive blue K-3R dye concentration. As can be seen from Figure 7, when the dye concentration was 0.5% and 3.0%, the peaks at 1658 cm^−1^ and 3324 cm^−1^ tended to be gentle and a new peak at 2360 cm^−1^ was produced. This showed that there was a chemical bond of –CN due to the reaction of carbonyl groups in *F. mandshurica* and –NH_2_ groups in reactive blue K-3R dye molecules [23,24].

### 3.4. Properties of Waterborne Coating on Surface of Dyed F. mandshurica Veneer

**Figure 8:**
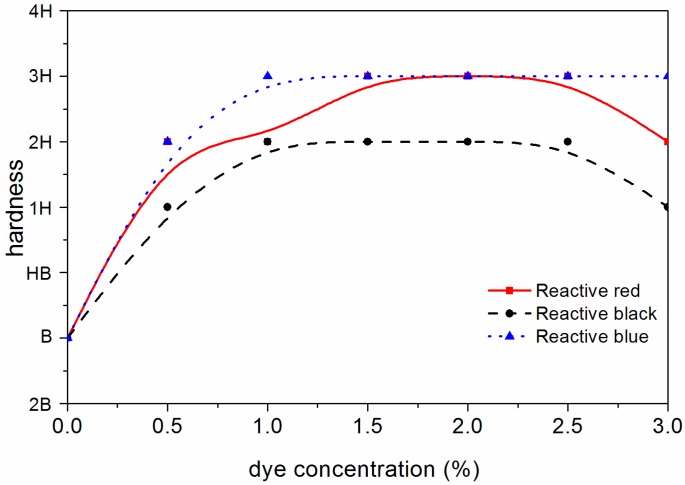
Variation trend of waterborne coating hardness on the *F. mandshurica* veneer after dyeing.

**Figure 9:**
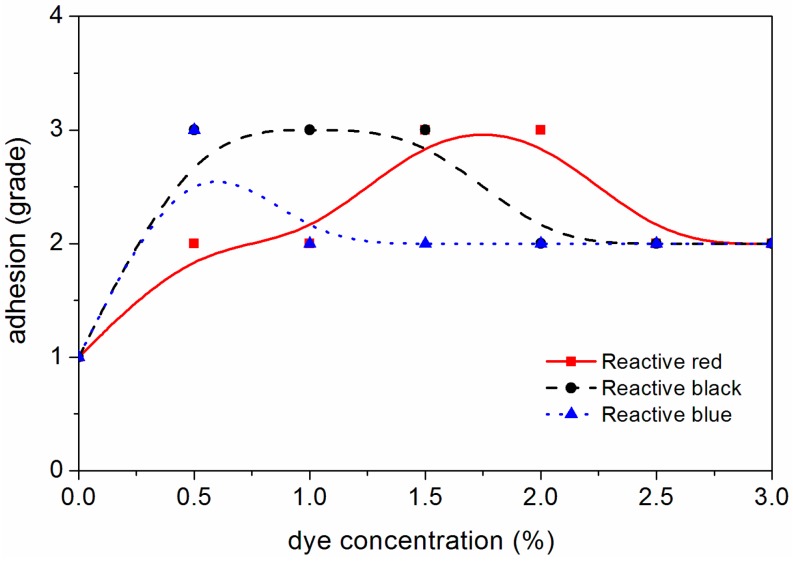
Variation trend of waterborne coating adhesion on the *F. mandshurica* veneer after dyeing.

**Figure 10:**
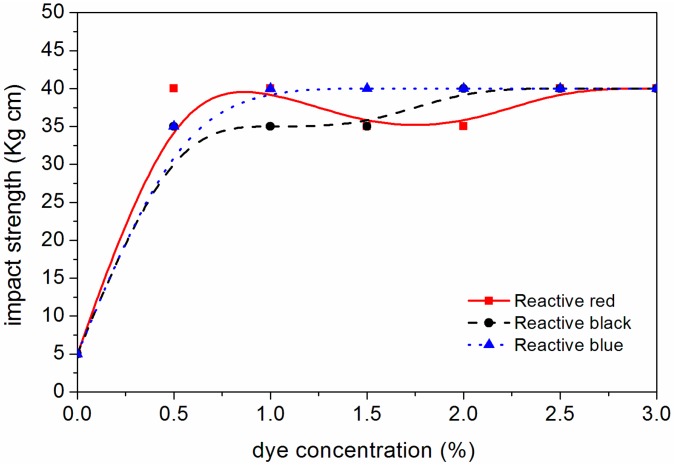
Variation trend of waterborne coating impact strength on the *F. mandshurica* veneer after dyeing.

**Figure 11:**
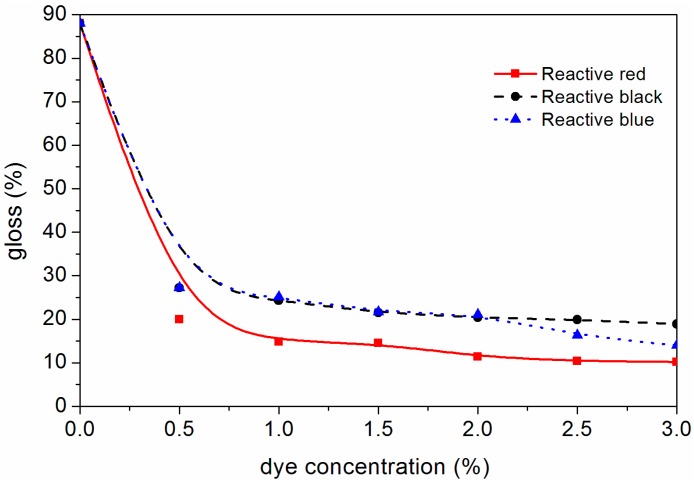
Variation trends of gloss of waterborne coating on the *F. mandshurica* veneer after dyeing.

The changes of different mechanical properties of the waterborne coating on the surface of dyed *F. mandshurica* veneer are shown in Figure 8, Figure 9, Figure 10 and Figure 11. It can be seen that when the concentration of reactive red dye increased from 0 to 2.0%, the waterborne coating hardness increased from B to 3H, the adhesion decreased from level 1 to level 3, the impact strength increased from 5.0 kg·cm to 35.0 kg·cm and the gloss of waterborne coating decreased from 88.0% to 11.4%. When the active black dye concentration increased from 0 to 1.0%, the waterborne coating hardness increased from B to 2H, the adhesion decreased from level 1 to 3, the impact strength increased to 35 kg·cm and the gloss of waterborne coating decreased from 88.0% to 24.3%. When the activated blue dye concentration increased from 0 to 1.0%, the waterborne coating hardness increased from B to 3H, the adhesion decreased from level 1 to level 2, the impact strength increased to 40 kg·cm and the gloss of waterborne coating decreased from 88.0% to 25.1%. According to the general requirements of the national standard for wooden furniture [25], the gloss of the waterborne coating on the surface of dyed *F. mandshurica* veneer is matte [26]. With further increases in the concentration of reactive dyes, changes in hardness, adhesion and impact resistance changed very little. Compared with undyed *F. mandshurica* veneer, the hardness of the waterborne coating increased, the adhesion decreased and the impact strength increased. This may be due to the reaction between *F. mandshurica* veneer and reactive dyes that changed the characteristics of the substrate and changed the performance of surface waterborne coatings.

## 4. Conclusions

The properties of the reactive brilliant red X-3B, black KN-B and blue K-3R dyes for dyeing of *F. mandshurica* veneer depend largely on the dye concentration. The dye uptake and chromatic aberration of *F. mandshurica* veneer showed that the concentration of 0.5–1.0% was better. The chromatic aberration showed improved color when the dye concentration was 0.5–2.5%. After combining the dye uptake and chromatic aberration, 0.5–1.0% was considered the better value. Therefore, the optimum conditions in the orthogonal test showed that the dyeing parameters of the reactive brilliant red X-3B, black KN-B and blue K-3R dyes for *F. mandshurica* veneer were temperatures of 75 °C, a dye concentration of 0.5%–1.0%, a dyeing time of 60 min and bath ratio of 20:1. The dye uptake of brilliant red X-3B dye was 75.0–75.4%, which was higher than that of the other two dyes. The chromatic aberration was 53–59, which was larger and looked more obviously dyed. During the dyeing process of the reactive brilliant red X-3B, reactive black KN-B and reactive blue K-3R dyes for *F. mandshurica* veneer, the physical and chemical reactions were included in two forms and when the dye concentration was increased in the experimental test range, the reaction between the reactive dye and *F. mandshurica* veneer was more obvious. After dyeing with reactive brilliant red X-3B, reactive black KN-B and reactive blue K-3R, the hardness of the waterborne coating on the surface of *F. mandshurica* veneer was increased, adhesion was decreased, impact strength was improved and gloss decreased to the matte level, all positive indications concerning the application of waterborne coating on the surface of dyed *F. mandshurica* veneer.

## Figures and Tables

**Table 1:** L_4_ (2^3^) results of dye uptake by *F. mandshurica* veneer.

Sample	Dye concentration (%)	Bath ratio	Dyeing time (min)	Dye uptake_Red_ (%)	Dye uptake_Black_ (%)	Dye uptake_Blue_ (%)
1_Red_, 1_Black_, 1_Blue_	1.0	20:1	60	75.0	50.0	66.0
2_Red_, 2_Black_, 2_Blue_	1.0	30:1	120	53.0	36.0	44.0
3_Red_, 3_Black_, 3_Blue_	5.0	20:1	120	8.4	22.0	42.0
4_Red_, 4_Black_, 4_Blue_	5.0	30:1	60	3.6	1.8	33.0
Range_Red_	58.0	13.4	8.6			
Range_Black_	31.1	17.1	3.1			
Range_Blue_	17.5	15.5	6.5			
Variance_Red_	3364.0	179.6	74.0			
Variance_Black_	967.2	292.4	9.6			
Variance_Blue_	306.3	240.3	42.3			

1_Red_, 2_Red_, 3_Red_ and 4_Red_ refer to the 1–4 samples of reactive brilliant red X-3B dye. 1_Black_, 2_Black_, 3_Black_ and 4_Black_ refer to 1–4 samples of reactive black KN-B dye. 1_Blue_, 2_Blue_, 3_Blue_ and 4_Blue_ refer to 1–4 samples of reactive blue K-3R dye. Range_Red_, Range_Black_ and Range_Blue_ are the range of reactive brilliant red X-3B dye, reactive black KN-B dye and reactive blue K-3R dye, respectively. Variance_Red_, Variance_Black_ and Variance_Blue_ are the variance of reactive brilliant red X-3B dye, reactive black KN-B dye and reactive blue K-3R dye, respectively. Dye uptake_Red_, Dye uptake_Black_ and Dye uptake_Blue_ are the dye uptake of reactive brilliant red X-3B dye, reactive black KN-B dye and reactive blue K-3R dye, respectively.

**Table 2:** L_4_ (2^3^) orthogonal test of chromatic aberration with reactive brilliant red.

Sample	L	a	b	L’	a’	b’	ΔL	Δa	Δb	ΔE
1_Red_	75.45	+5.79	+20.86	37.08	+45.75	+8.39	−38.37	+39.96	−12.47	56.79
2_Red_	76.77	+5.35	+20.11	33.09	+41.59	+13.26	−43.68	+36.24	−6.85	57.17
3_Red_	74.30	+5.97	+21.15	30.33	+36.21	+12.97	−43.97	+30.24	−8.18	53.99
4_Red_	75.14	+5.95	+19.92	30.87	+38.00	+13.71	−44.27	+32.05	−6.21	55.01

**Table 3:** L_4_ (2^3^) orthogonal test of chromatic aberration with reactive black.

Sample	L	a	b	L’	a’	b’	ΔL	Δa	Δb	ΔE
1_Black_	75.79	+6.15	+21.01	20.82	+1.73	−2.57	−54.97	−4.42	−23.58	59.98
2_Black_	74.61	+5.99	+20.49	22.41	+1.28	−2.65	−52.20	−4.71	−23.14	57.29
3_Black_	74.90	+5.87	+20.06	20.29	+4.29	−0.36	−54.61	−1.58	−20.42	58.32
4_Black_	74.46	+5.70	+20.07	20.45	+4.03	−0.61	−54.01	−1.67	−20.68	57.86

**Table 4:** L_4_ (2^3^) orthogonal test of chromatic aberration with reactive blue.

Sample	L	a	b	L’	a’	b’	ΔL	Δa	Δb	ΔE
1_Blue_	75.29	+5.99	+21.61	23.08	+1.26	−5.91	−52.21	−4.73	−27.52	59.21
2_Blue_	74.46	+5.97	+20.07	22.98	+1.23	−5.44	−51.48	−4.74	−25.51	57.65
3_Blue_	74.91	+6.03	+20.83	19.84	+1.93	−3.40	−55.07	−4.10	−24.23	60.30
4_Blue_	75.71	+5.81	+21.71	21.04	+2.21	−3.64	−54.67	−3.60	−25.35	60.37

**Table 5:** Assignment of peaks.

Peak (cm^−1^)	Assignment
3380	–OH stretching vibration
2360	–CN stretching vibration
1658	N–H–O absorption
1171	C–O–C bond absorption
3324	absorption of N–H
1730	carbonyl groups
